# The impact of different feeds on DNA methylation, glycolysis/gluconeogenesis signaling pathway, and gene expression of sheep muscle

**DOI:** 10.7717/peerj.13455

**Published:** 2022-05-26

**Authors:** Feng Song, Zaccheaus Pazamilala Akonyani, Ying Li, Deqiqige Su, Lantuya Wu, Yue Pang, Sile Hu, Dubala Wu, Chun Li, Ding Yang, Jianghong Wu

**Affiliations:** 1 College of Animal Science and Technology, Inner Mongolia Minzu University, Tongliao, Inner Mongolia, China; 2 College of Life Sciences and Food Engineering, Inner Mongolia Minzu University, Tongliao, Inner Mongolia, China; 3 Inner Mongolia Academy of Agricultural & Animal Husbandry Sciences, Hohhot, Inner Mongolia, China

**Keywords:** cAMP pathway, DNA methylation, Glucose metabolism, Glycolysis/Gluconeogenesis, Whole-genome bisulfite sequencing

## Abstract

DNA methylation is an important epigenetic regulatory form that regulates gene expression and tissue development. This study compared the effects of high fiber, low protein (HFLP) and low fiber, high protein (LFHP) diets on the DNA methylation profile of twin lambs’ muscles, their effect on glycolysis/gluconeogenesis and related pathways by transcriptome and deep whole-genome bisulfite sequencing (WGBS). Results identified 1,945 differentially methylated regions (DMRs) and 1,471 differentially methylated genes (DMGs). Also, 487 differentially expressed transcripts belonging to 368 differentially expressed genes (DEGs) were discovered between the twin lambs under different diets. Eleven overlapped genes were detected between the DEGs and the DMGs. *FKBP5* and *FOXO1* were detected to be significantly different. The *FOXO1* regulated cAMP and the glycolysis/gluconeogenesis pathways. The glycolysis/gluconeogenesis, and the FOXO pathways were significantly enriched. The expressions of *HOMER1* and *FOXO1* in the HFLP group were significantly higher than those in the LFHP group. There is a significant correlation between the upregulated gene expression and hypomethylation of *HOMER1* and *FOXO1* gene in HFLP group. The results showed that *FOXO1* induces *PDK4* expression in muscle while regulating *FKBP5* activity, which stimulates glucose production by activating specific gluconeogenesis target genes. The *FOXO1* was able to regulate the glucose metabolism, the cAMP and the occurrence of glycolysis/gluconeogenesis pathways. This study showed that feed type can affect the methylation levels of the glycolysis related gluconeogenesis genes and interaction pathways, providing new ideas for a better understanding of the regulation of muscle energy metabolism and feed development.

## Introduction

Epigenetic regulation is a key link in the interaction between nutrition and the host genome. This provides more variable space for host gene expression (Anderson, Sant & Dolinoy, 2012). Sheep are an essential breed of livestock that offer humans a huge amount of meat, fur, and milk. Twins are valuable materials for epigenetic research (Jiang et al., 2014). Their genetic information is extremely consistent. Meat quality factors such as muscle fatty acid profile, tenderness, share force, and other indicators can be affected by different pastures and nutrition in twin lambs. Environmental factors affect the expression of target genes through DNA methylation levels, which in turn affects the formation of their phenotype (Wang et al., 2015).

DNA methylation plays an important role in the control of gene expression and tissue development by genetically altering gene expression and function without altering the DNA sequence (Yen et al., 2016). The occurrence of DNA methylation can inhibit the transcriptional activity of genes and reduce expression levels, while demethylation is reactivated to increase expression levels (Moore, Le & Fan, 2013). DNA methyltransferase (DNMT) is an enzyme that adds methyl to cytosine to produce 5-methylcytosine (5mC). The 5mC blocks the binding of transcription factors, which leads to the inhibition of gene expression, the effect on metabolism and genome, how the genome is based on different metabolites, as well as how metabolic networks regulate changes at the genome level (Rauch & Mandrup, 2016).

Previous research has shown that introducing methylhistamine to a high-fiber diet can enhance stomach acid secretion and boost cAMP levels. Protein kinase A phosphorylates pyruvate kinase in response to an increase in cAMP concentration, and its decreased activity inhibits glycolysis in lamb muscles. Furthermore, a high protein diet can enhance muscle differentiation and growth by increasing taurine and adenine hydrochloride levels (Wu et al., 2020).

The gluconeogenic enzymes phosphoenolpyruvate carboxykinase (PEPCK), fructose 1,6-bisphosphatase (Fru-l,6-P2ase), and possiblyglucose-6-phosphatase are activated by a combination of increased intracellular cAMP and decreased plasma insulin (G-6-Pase). Transcript of the genes encoding the glycolytic enzymes glucokinase (GK), 6-phosphofructo I-kinase (6-PF-I-K), and pyruvate kinase (PK), as well as the bifunctional enzyme, is reduced. The increased levels of cAMP must be reduced initially, followed by a rise in Fru-2,6-P2. Insulin must additionally suppress the PEPCK and Fru-l,6-P2ase while inducing the mRNAs for GK, PK, and 6-PF-2-K/Fru-2,6-P2ase for complete restoration. It is obvious that the amount of carbon flux in the glycolytic/gluconeogenetic pathway is influenced by a number of complex parameters, the contribution of which varies depending on the animal’s nutritional and hormonal status (Pilkis & Granner, 1992).

Despite the fact that relevant studies have been published in recent years, the regulating mechanism remains unknown. Therefore, it is crucial to understand the effects of different diets on muscle methylation levels, cAMP and glycolysis/gluconeogenesis signaling pathways. In this study, whole genome methylation and transcriptome sequencing techniques were used to investigate the effects of different diets on DNA methylation, cAMP, glycolysis/gluconeogenesis signaling pathways, and their expression in the muscles of twin lambs. The advancement of contemporary animal husbandry has given rise to new theories that can aid in the understanding of the phenotypic differences that arise during the domestication of animals.

## Materials and Methods

Pellet feed preparation, DNA library preparation and sequencing, and gene expression analysis of longissimus dorsi (LD) muscle were all consistent with our previous study (Wu et al., 2020). Two pellet feeds were prepared, and their nutritional contents measured: one with 25% concentrate and 75% ceratoides hay, comprising the HFLP group, and the other with 25% concentrate and 75% alfalfa hay, comprising the LFHP group.

### Animal experiments and sampling

To reduce the effect of genetic background, four pairs of 3-month-old Chinese Sunit lamb (*Ovis aries*) female twins weighing 24 ± 2.3 kg were used in the pairing experiments. They were randomly divided into two groups and fed for 110 days either with HFLP or LFHP pellets. The twin lambs were selected from Siziwangqi, Jining, China. All the lambs were fed during the test period. Immunization, deworming, bathing, disease prevention and control were carried out as usual, and water was given *ad libitum*. The two groups were kept in a small corral. After a fasting period, the lambs were slaughtered in a local abattoir, and were euthanised through captive bolt stunning, followed by exsanguination. After the slaughter, two pieces of LD muscle between the 12th and 13th ribs were sampled from each individual and preserved in a nitrogen canister.

### WGBS and identification of DMRs

The Illiumina HiSeqTM2500 platform was used to sequence libraries (Biomarker Technologies, Beijing, China). A base detection was done to convert the peak signal to sequence data, and the original reading was quality filtered to obtain a pure reading. The reading of the 3′ junction subsequence was first trimmed, and the values that had more than 10% unknown base (N) and low quality (more than 50% PHRED score ≤5 base) were discarded. Also, the Q30 and CG contents were estimated at the same time. The clean reads were aligned to the sheep reference genome (Oar_v4.0) (https://www.sheephapmap.org/), and the bisulfite mapping of methylation sites was performed using Bismark software. The sequencing depth and coverage were assessed after the duplicate reads were aligned with the same region of the genome. The bisulfite conversion rate is the proportion of methylated clean reads in the genome compared to the total number of clean reads. To confirm C-site methylation by screening conditions for ≥4× and false discovery rate (FDR) <0.05, the binomial distribution test for each C site was used (Fan et al., 2020).To identify DMR, the model of the estimated methylation level was referenced. All C sites with read coverage >10× were used for DMR analysis with MOABS (Sun et al., 2014). There were at least three differential methylation sites, and the minimum methylation level difference was 0.1 (CG type was 0.2) using Fisher’s exact test, *P* < 0.1.

### Functional enrichment analysis

To annotate gene functions, DMGs were compared with the functional databases (GO, KEGG, and COG) by BLAST. The WALLSEius non-center hypergeometric distribution of the GOseq R package (Young et al., 2010) was used for DMGs GO enrichment analysis. In KEGG pathway analysis, the KOBAS software was used to determine the relevance of DMG enrichment (Mao et al., 2005). An enrichment analysis of the differential genes was performed with ClueGO (Bindea et al., 2009), which is a plugin of Cytoscape (Shannon et al., 2003). The GO annotation was used to classify the DMGs and DEGs into cellular components, molecular functions, and biological processes.

## Results

### DNA methylation mapping and patterns

**Figure 1:**
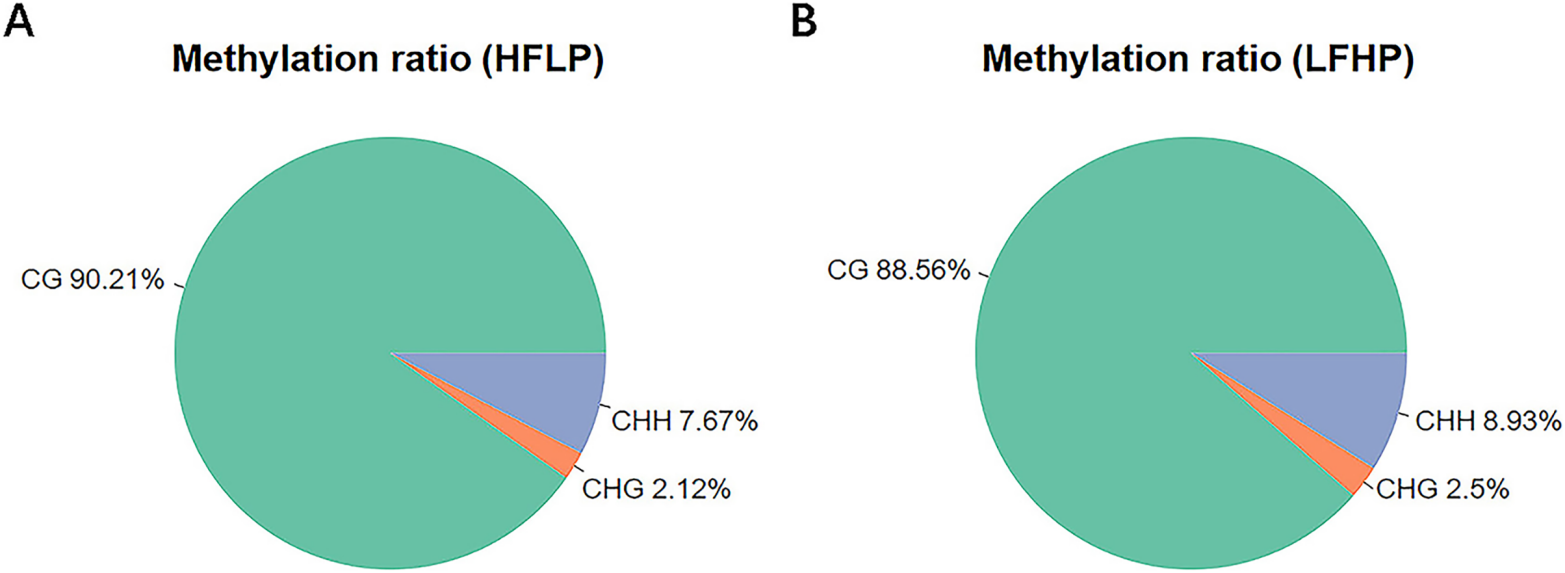
Analysis of methylation preferences. The average ratio of DNA methylation types within the HFLP and LFHP genomes of Sunit twin lambs. The green, red, and blue colors depict methylated (CG, CHG, and CHH) respectively, where H = A/C/T.

The entire DNA methylation analysis of the LD muscle of the twin lambs was carried out by WGBS with 10× genome coverage and >99% conversion rate. Approximate values of 293.17 M clean reads were uniquely mapped to produce the twin lamb HFLP methylome, and 290.10 M clean reads for the production of the twin lamb LFHP methylome. The quality of sequencing data is detailed (Table 1). Approximately a total of 3.0% of all genomic sites were methylated in every group (Table 1). Methylation in the twin lambs was detected to appear in three contexts of the sequence, namely, CG, CHG, and CHH (where H = A/C/T). However, their methylation levels were different. The entire genome-wide mC levels of 90.21% (CG), 2.12% (CHG), and 7.67% (CHH) within the HFLP specimen (Fig. 1A), and 88.56% (CG), 2.50% (CHG), and 8.93% (CHH) within the LFHP specimen (Fig. 1B). These contexts of sequence were almost the same in every group.

**Table 1:** Data statistics of entire DNA bisulfite sequencing reads for twin lambs.

Samples	Clean base (Gb)	Clean reads(M)	GC (%)	Q30 (%)	Unique mapped (M)	Mapped (%)	Conversion rate (%)	Total mC (%)
HFLP	85.89	293.17	21.85	91.20	196.45	67.01	99.61	3.00
LFHP	84.98	290.10	20.88	91.16	193.28	66.63	99.48	3.00

**Note:**

Clean Base (Gb), base after filtration; Clean Reads, the number of reads after filtering; GC (%), the percentage of G and C type bases in the total bases; Q30 (%), the percentage of bases with a mass value greater than or equal to 30 in the total base number; Unique mapped, the number of clean reads uniquely mapped to the reference genome; Mapped (%), the percentage of clean reads matched to the reference genome relative to the total clean reads; Conversion Rate (%), the percentage of clean reads matched to the reference genome relative to the amount of methylation of the clean reads matched to the reference genome. Total mC (%), the number of clean reads matched to the reference genome relative to the amount of methylated cytosine within the clean reads matched to the reference genome.

### Characterization of DMRs and gene annotation

**Figure 2:**
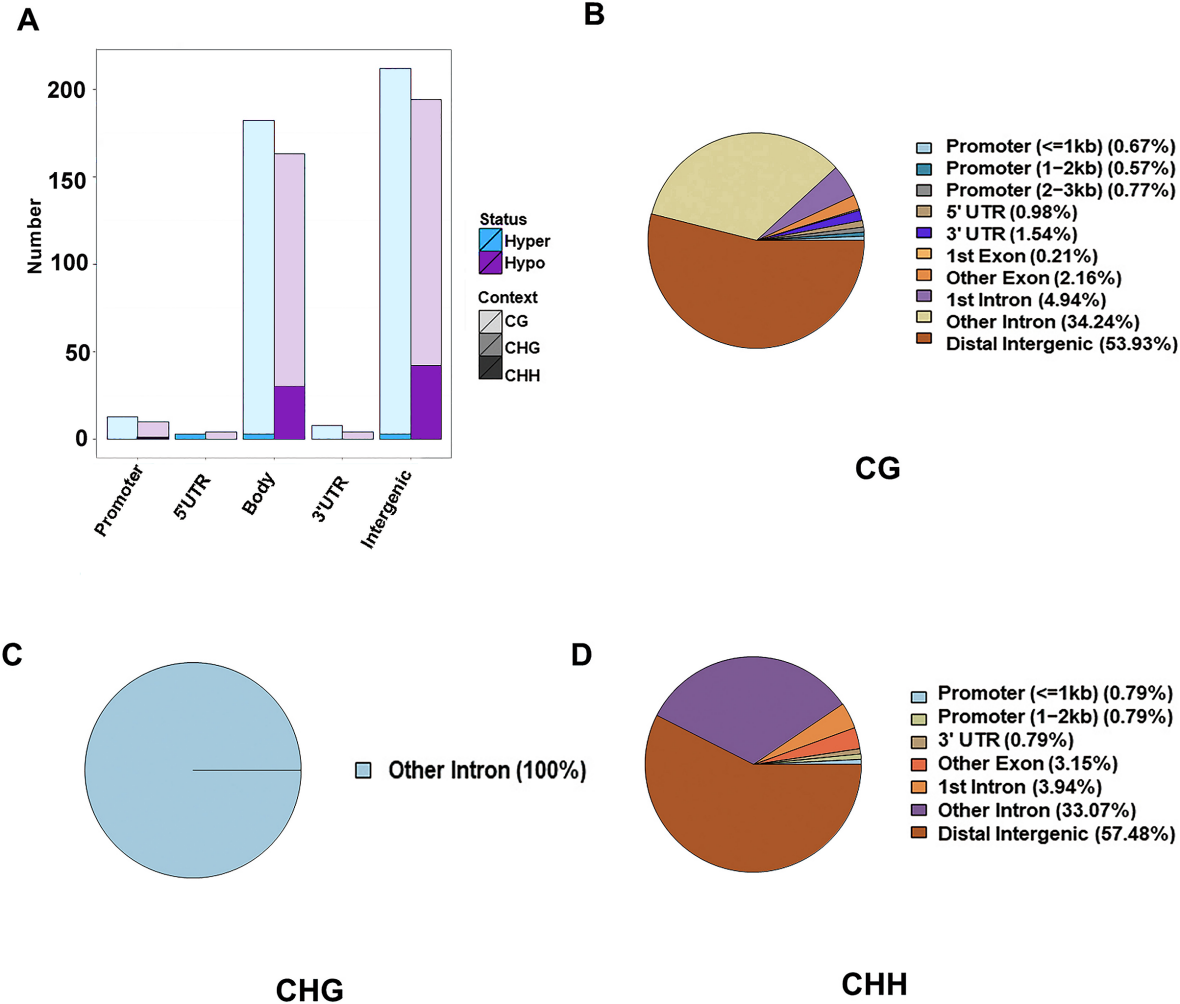
Identification of DMRs between the HFLP and LFHP twin lamb muscle samples. (A) The number of DMRs between different methylation types. The abscissa is the functional region of the gene, and the ordinate is the corresponding DMR number. Different colors represent different methylation states, and different color gradients represent different methylation types. The pie charts display the distribution numbers of DMRs in distinct genomic regions in the CG (B), CHG (C), and CHH (D).

The DMR were determined within the two samples, and were annotated into the gene functional regions based on varied methylation contexts. A total of 1,945 CG DMRs, 127 CHH DMRs, and three CHG DMRs were detected. Most of these CG DMRs were detected in the distal intergenic region, with only 19 and 30 found in the 5′UTR and 3′UTR, respectively. CHG DMRs were all located in other introns, and CHH DMRs were mainly located in the distal intergenic regions. Of the 1,945 CG DMRs, a total of 1,022 were hypermethylated and 923 were hypomethylated. Out of the 127 CHH DMRs, a total of 105 were hypomethylated and 22 were hypermethylated. However, all the three CHG DMRs were hypomethylated. Except for those in the distal intergenic regions, the ratio of DMRs in introns was the greatest for all methylation types. A total of 1,471 CG DMGs, 106 CHH DMGs, and three CHG DMGs were annotated (Fig. 2).

### Function enrichment analysis of DMGs

Analysis of the COG, GO and KEGG pathway databases was carried out, and 1,471 DMGs were found in the DMRs. The COG analyses showed that DMGs were enriched in general function prediction only, mostly for CG. The GO analysis for the CG context showed that DMGs were substantially enriched in the classification of negative regulation of transcription from RNA polymerase II promoter, cell junction, and calcium ion binding. The 20 most substantially uniquely enriched muscle development associated GO terms for DMGs between HFLP and LFHP LD samples are displayed. The KEGG analysis for the CG context also discovered that the DMGs were greatly enriched in pathways in cancer, axon guidance, MAPK, and Wnt signaling pathways. The results revealed that DMGs, which are affected by DNA methylation, can influence muscle development, glucose and fatty acid metabolism (Table S1, Fig. S1).

**Figure 3:**
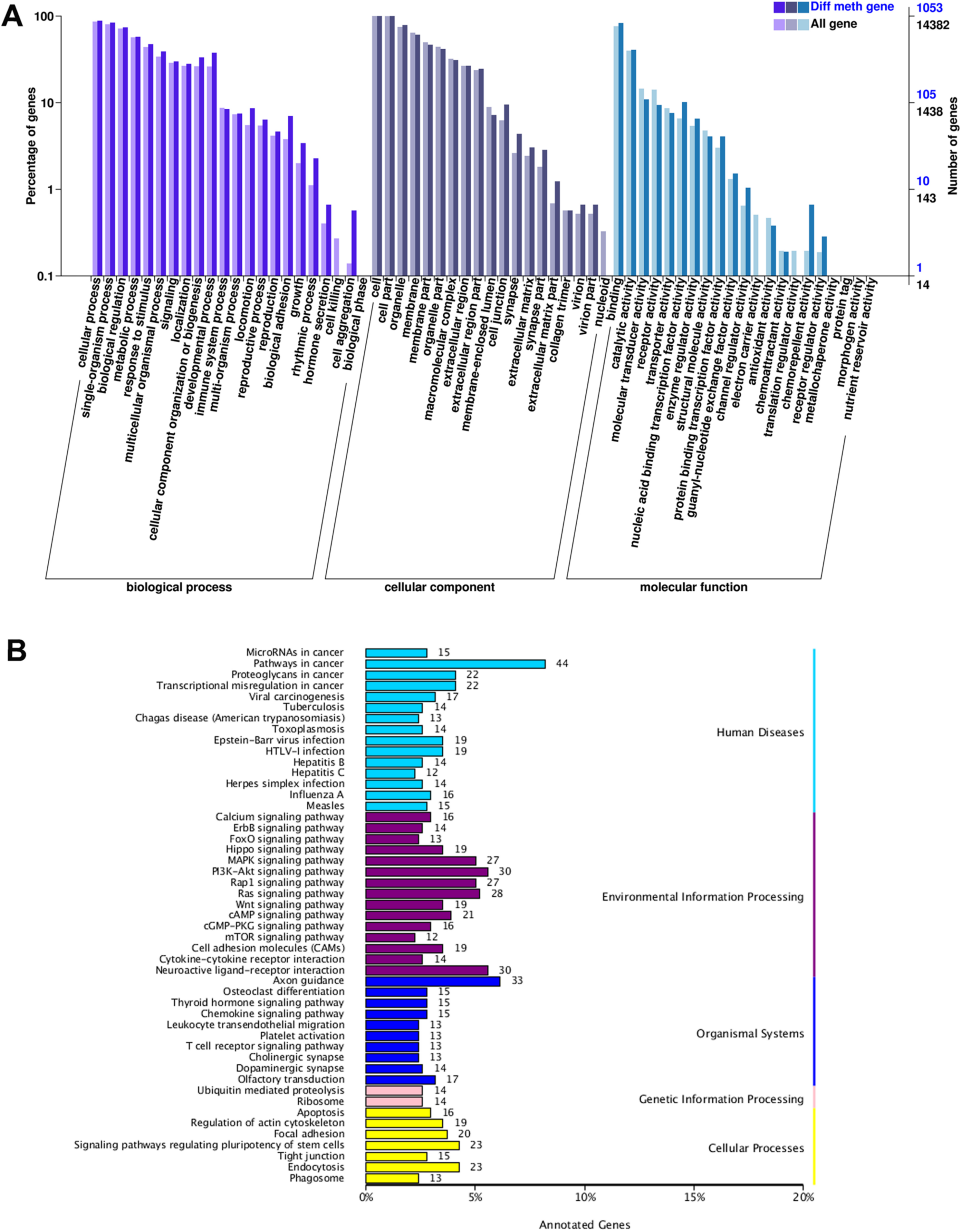
GO and KEGG enrichment analysis of DMGs. (A) The GO enrichment analysis of DMGs, and (B) the KEGG enrichment analysis of DMGs.

According to GO function classification, DMGs were divided into molecular function, cellular components, and biological processes (BP). The cAMP was enriched in BP by five genes (*LOC101106702*, *GNAI1*, *CFTR*, *PIK3CG*, and *SOX9*), and the cellular processes, single biological processes, metabolic processes, and biological regulation. Increasing *LOC101106702* promoted positive regulation of BP cAMP-dependent protein kinase activity. *GNAI1* can regulate cAMP-mediated signaling pathway. *CFTR* and *PIK3CG* are responsible for cell response to cAMP, and *SOX9* can induce cAMP-mediated signaling pathway (Fig. 3A).

The KEGG pathway enrichment analysis of DMGs involved a total of 49 functions. It was found that genes with differential DNA methylation were enriched when human diseases, environmental information processing, organismal systems, genetic information processing, and cellular processes were included in the KEGG enrichment analysis. In the process of environmental information processing, the cAMP pathway was significantly enriched, including 21 genes (*MAPK10*, *DRD5*, *DRD1*, *MLLT4*, *SOX9*, *LOC101106702*, *AKT3*, *RRAS2*, *HHIP*, *SSTR1*, *RELA*, *GRIN3A*, *FSHR*, *PPP1R12A*, *LOC101104054*, *ATP2B1*, *GRIN2B*, *GNAI1*, *PIK3CG*, *CFTR*, and *VAV3*). The glycolysis/gluconeogenesis pathway was significantly enriched, including four genes (*HKDC1*, *PDHA2*, *PGM2*, and *PDHA1*). The FOXO pathway was significantly enriched, including 13 genes (*MAPK10*, *HOMER1*, *FOXO1*, *AKT3*, *FOXG1*, *BCL6*, *LOC101103187*, *EGFR*, *CHUK*, *IRS4*, *GADD45A*, *PIK3CG*, and *S1PR1*) (Fig. 3B).

### Gene expression of LD muscle under two feeds

The Illumina Hi-Seq 2500 was used to sequence 8 LD muscle samples (four repetitions per set), and 22.5 to 28.5 million 125-bp clean paired-end reads were generated. The statistical power of this experimental design, calculated in RNASeqPower (Hart et al., 2013) is 0.9917946 (rnapower (depth = 23.68, cv = 0.4, effect = 4, *n* = 4, alpha = 0.05)). A reference transcriptome using obtained RNA-seq reads was reconstructed by the Trinity software. There were 347,335 transcripts in the reference transcriptome, with an average length of 2,166.29 bp. The edgeR package in R was used to analyze the DEGs between the twin lambs under different diets. With *P* = 0.0001 and a false discovery rate (FDR) of 0.05, 487 differentially expressed transcripts belonging to 368 DEGs were discovered, including 67 novel genes.

A KEGG pathway enrichment analysis on the DEGs in the muscles of twin lambs under different diets (Fig. S2) was performed, and the *PDK4* gene was determined to play a vital role in the regulation of glucose and fatty acid metabolism. The key gene, *PDK4* (Log CPM = 11.584, *P* = 0.000, and FDR = 0.016), was found to exhibit the greatest expression levels in LD muscle samples under the feed conditions.

### Joint analysis of DMGs and DEGs in LD muscle

**Figure 4:**
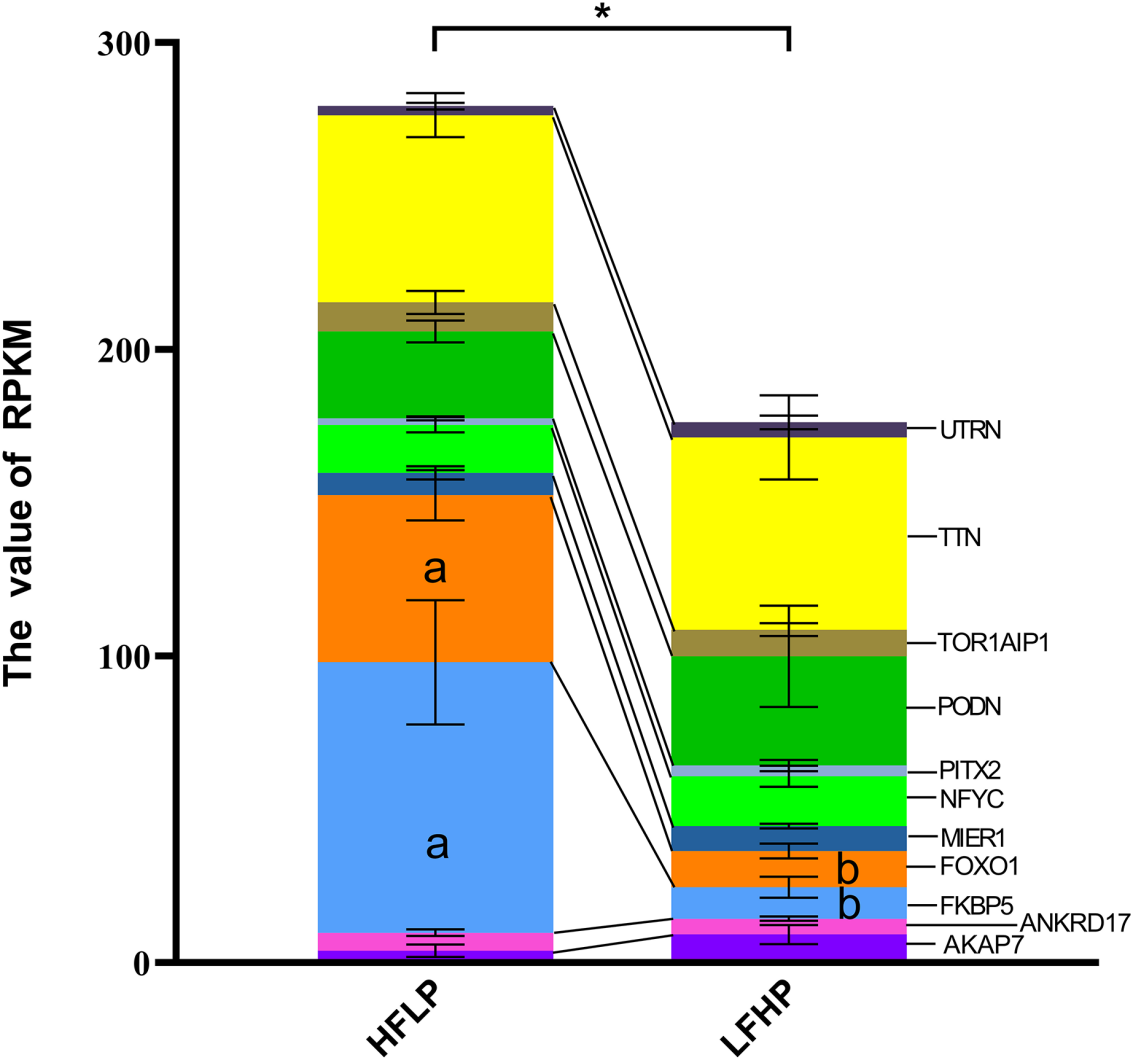
Gene expression levels between the DEGs and the DMGs. The ordinate denotes the reads per kilobase million (RPKM), and the abscissa denotes the two feed groups. The HFLP represents the ceratoid feed group, and the LFHP represents the alfalfa feed group. The lowercase letters a and b represent significant differences. The asterisk (*) represents a significant difference between the two groups.

It was noticed that 1,471 DMGs were found between the two groups (LFHP and HFLP). A total of 11 overlapping genes (*AKAP7*, *ANKRD17*, *FKBP5*, *FOXO1*, *MIER1*, *NFYC*, *PITX2*, *PODN*, *TOR1AIP1*, *TTN*, and *UTRN*) were detected between DEGs and DMGs (Fig. 4). Six of the 11 genes were hypermethylated (*AKAP7*, *FKBP5*, *MIER1*, *PITX2*, *PODN*, and *UTRN*), while the other five (*ANKRD17*, *FOXO1*, *NFYC*, *TOR1AIP1*, and *TTN*) were hypermethylated in the LFHP group compared with the HFLP group. Among the 11 genes, seven genes (*AKAP7*, *MIER1*, *NFYC*, *PITX2*, *PODN*, *TTN*, and *UTRN*) were expressed at higher levels in the LFHP diet than in the HFLP diet. Also, four genes (*ANKRD17*, *FKBP5*, *FOXO1*, and *TOR1AIP1*) had a higher expression level in the HFLP diet than those in the LFHP diet. Expression levels in the HFLP diet group were significantly higher than in the LFHP diet group (*P* = 0.02). The expression levels of *FKBP5* and *FOXO1* genes were significantly higher in the HFLP diet than in the LFHP diet (*P* = 0.03, *P* = 0.01). Of these genes, *FKBP5* (Log CPM = 8.101, *P* = 0.000, and FDR = 0.000), which plays a role in the intracellular trafficking of heterooligomeric forms of steroid hormone receptors maintaining the complex in the cytoplasm when unliganded, had high expression levels in the two diets. *FOXO1* (Log CPM = 4.461, *P* = 0.000, and FDR = 0.025) is involved in the regulation of gluconeogenesis by regulation of transcription from the RNA polymerase II promoter.

### cAMP pathway analysis

**Figure 5:**
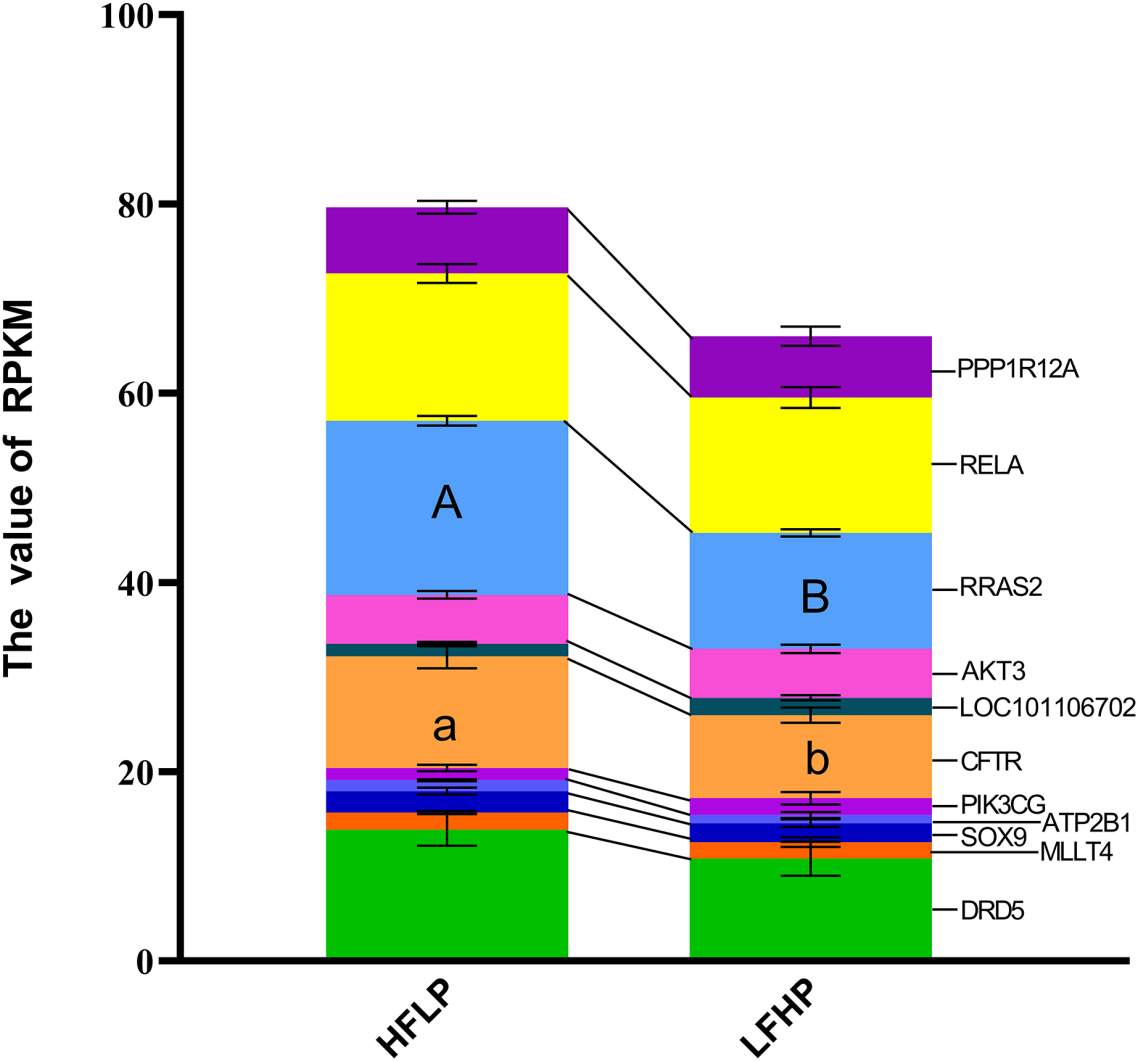
Effect of HFLP and LFHP diet on gene expression related to the cAMP pathway. The ordinate denotes the reads per kilobase million (RPKM), and the abscissa denotes the two feed groups. The HFLP represents the ceratoid feed group, and the LFHP represents the alfalfa feed group. The letters a and b represent significant differences, while A and B represent extremely significant differences.

A KEGG pathway enrichment analysis was executed using ClueGO to determine the functions of identified DMGs. A total of 274 enriched pathways were detected, including the cAMP signaling pathway (Table S1). A total of 21 DMGs genes were detected in the cAMP signaling pathway. From the data, 12 genes *(MAPK10*, *DRD1*, *MLLT4*, *LOC101106702*, *RRAS2*, *HHIP*, *RELA*, *PPP1R12A*, *LOC101104054*, *GNAI1*, *PIK3CG*, and *VAV3*) were hypomethylated, eight of the genes (*DRD5*, *SOX9*, *AKT3*, *GRIN3A*, *FSHR*, *ATP2B1*, *GRIN2B*, and *CFTR*) were hypermethylated in the LFHP group compared with the HFLP group. These genes (*MAPK10*, *DRD5*, *DRD1*, *MLLT4*, *SOX9*, and *AKT3*) are mostly related to many functions, such as glucose metabolism, lipid metabolism, production of insulin and leptin signaling, and insulin secretion by regulating the ERK/P38 signaling pathway. The cAMP activity in twin lambs fed with the HFLP diet was higher than that in twin lambs fed with the LFHP diet. In the HFLP diet, nine genes had a higher expression than those in the LFHP diet. However, two genes in the LFHP diet had a higher expression than in the HFLP group (Fig. 5). The expression of the *CFTR* gene in HFLP was significantly higher than that in the LFHP diet (*P* = 0.1), and the expression level of *RRAS2* was detected to have an extremely significant difference in the HFLP diet than in the LFHP diet (*P* = 0.01). Based on the cAMP signaling pathway activity, the *RRAS2* was extremely increased by the HFLP diet. The *RRAS2*, which is one of the Ras families, is capable of controlling insulin and glucose metabolism. Finally, the *CFTR*, which was significantly increased by the HFLP diet, is responsible for the movement of ions such as chloride and phosphorite ions across the cell, and could play a role by impairing insulin secretion (Fig. 5).

### Glycolysis/gluconeogenesis pathway

**Figure 6:**
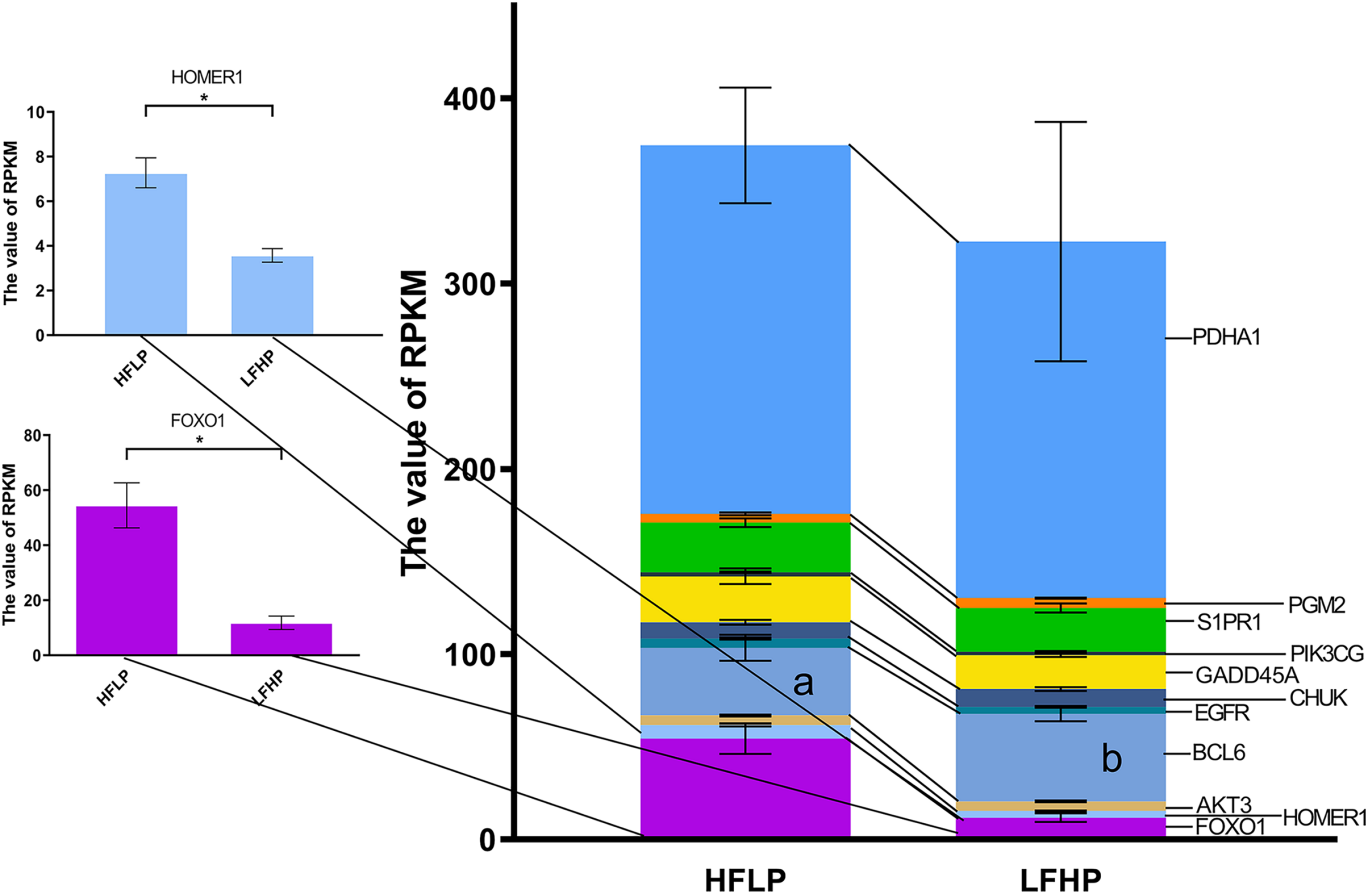
Effect of HFLP and LFHP diet on gene expression related to the glycolysis/gluconeogenesis and FOXO pathway. The ordinate denotes the reads per kilobase million (RPKM), and the abscissa denotes the two feed groups. The lowercase letters a and b represent significant differences. The asterisk (*) represents a significant difference between the two groups.

Further analysis of the functional classification of DMGs using ClueGO software revealed that the glycolysis/gluconeogenesis pathway and the FOXO pathway were significantly enriched, and the FOXO pathway was the key pathway regulating gluconeogenesis. Of the 17 genes in both pathways, eight genes were hypermethylated (*HOMER1*, *FOXO1*, *AKT3*, *FOXG1*, *EGFR*, *CHUK*, *S1PR1*, and *PDHA1*) and eight genes were hypomethylated (*MAPK10*, *BCL6*, *IRS4*, *GADD45A*, *PIK3CG*, *HKDC1*, *PDHA2*, and *PGM2*) in the LFHP group compared with the HFLP group. Eight genes were highly expressed in the HFLP group than in the LFHP group (*HOMER1*, *FOXO1*, *AKT3*, *EGFR*, *GADD45A*, *PIK3CG*, *S1PR1*, and *PDHA1*), while three genes were highly expressed in the LFHP group than in the HFLP group (*BCL6*, *CHUK*, and *PGM2*). The HFLP group had considerably higher expression of *HOMER1* and *FOXO1* than the LFHP group (*P* = 0.01). There is a significant correlation between the upregulated gene expression and hypomethylation of *HOMER1* and *FOXO1* gene in HFLP group (Fig. 6).

## Discussion

In this study, the WGBS was used to investigate the DNA methylation profiles of the genome in the LFHP and HFLP diets of twin lambs to discover the relationship between DNA methylation, glycolysis/gluconeogenesis, and related pathways of different levels of feed.

In this research, the *FOXO1* and *FKBP5* were identified in DMGs and DEGs among the 11 overlapped genes. *FOXO1* was highly expressed in the muscles of twin lambs fed with HFLP. A study showed that *FOXO1* is also part of a transcriptional interplay that regulates gluconeogenesis during prolonged periods of fasting (Kousteni, 2012). At the same time, *FOXO1* is increased in the muscles of diabetic patients (Stentz & Kitabchi, 2007). *PDK4* had the highest expression in the muscles of the HFLP- fed twin lambs. The *PDK4* is known to play an essential role in maintaining normal blood glucose levels and enhancing fat metabolism with regards to prolonged fasting (Andrews, 2004). *FOXO1* could increase the expression level of *PDK4* by directly binding to its promoter region. In type 2 diabetes, up-regulation of *PDK4* also inactivates PDC, which promotes gluconeogenesis and thereby contributes to the hyperglycemia characteristic of this disease (Jeoung & Harris, 2010). A study found that *FOXO1* induces *PDK4* expression in the context of muscle energy deprivation (Furuyama et al., 2003). Of the 11 overlapped genes, *FKBP5* had a high expression level in the HFLP diet. *FKBP5* expression is associated with insulin resistance, and its activity is regulated by *FOXO1* (Hou & Wang, 2012). Also, *FOXO1* and *FKBP5* act as glucocorticoids, which can stimulate glucose production by activating specific gluconeogenesis target genes (Wong et al., 2010).

*FOXO1* is an important downstream mediator of the insulin signaling pathway. cAMP-response element sites in the proximal promoter region of the *FOXO1* gene can activate *FOXO1* by acetylation (Wondisford et al., 2014). In this research, the cAMP signaling pathway *RRAS2* was extremely significantly elevated in HFLP, but *CFTR* was significantly elevated in HFLP diet expression. A study revealed that the Ras family can interact with the *MAPK* signaling pathway to modulate adipocyte insulin and glucose metabolism (Makdissy et al., 2015). The results showed that the expression of *CFTR* was low in the LFHP diet. The *CFTR* gene is a cAMP-regulated chloride channel expressed in the parietal membrane of various epithelial cells (Gregory et al., 1990). A study showed that the *FOXO1* gene as a potential regulator can regulate the glucose tolerance of *CFTR* (Montanini et al., 2016). A study suggests that the gene *CFTR*, which works as a glucose-sensing negative regulator of glucagon secretion in a cell, could lead to glucose intolerance in cystic fibrosis and other kinds of diabetes (Huang et al., 2017).

In this research, significant enrichment of the glycolysis/glycogenesis and FOXO pathways occurred, and FOXO is a key pathway regulating gluconeogenesis (Zhang et al., 2018). *PDHA1* was highly expressed in the muscles of twin lambs fed HFLP. The *PDHA1* gene is an important part of the pyruvate dehydrogenase complex, which catalyzes pyruvate decarboxylation and acts as a gate-keeper enzyme between glycolysis and the mitochondrial citric acid cycle. Inhibiting pyruvate dehydrogenase in cancer cells promotes the Warburg effect in cancer cells, making them more malicious (Kim et al., 2006). *FOXO1* is able to induce the up-regulation of *PDK4* in pyruvate dehydrogenase (Piao et al., 2013). *PGM2* is highly expressed in the muscles of twin lambs fed LFHP. A study showed that genes of the glycolysis pathway are of particular interest, among which *PGM2* and *PDHA1* are involved in glucose metabolism and are highly expressed in muscular young bulls (Bernard et al., 2009). The results showed that *HOMER1* and *FOXO1* were highly expressed in the muscles of HFLP-fed twin lambs. *HOMER1* is able to regulate muscle development, metabolism, and protein expression (Zimmermann, Neuhuber & Raab, 2017). Moreover, *HOMER1* is a highly expressed protein in hepatocellular carcinoma tissues and is a key gene associated with glycolysis (Jiang et al., 2019). *FOXO1* is one of the transcriptional interactions that regulates gluconeogenesis during prolonged fasting. Glucagon stimulates the gluconeogenic program by activating CREB-regulated transcriptional co-activator 2 (*CRTC2*), while a parallel reduction in the insulin signaling pathway increases gluconeogenesis gene expression through *FOXO1* activation (Kousteni, 2012).

## Conclusions

This study systemically showed the global DNA methylation pattern of twin lambs’ muscles associated with energy metabolism. The differences between the HFLP and LFHP diets in genomic DNA methylation were explained, as were the genes and pathways associated with glycolysis/gluconeogenesis pathways in the twin lambs. The results showed that the HFLP and LFHP diets significantly affected gene expression in DNA methylation, energy metabolism, and muscle glycolipid metabolism. The results also revealed that the global DNA methylation pattern of twin lambs’ muscles associated with energy metabolism between the HFLP and LFHP diets could provide new insights into a better understanding of the epigenetic regulation of sheep muscle development.

## Supplemental Information

10.7717/peerj.13455/supp-1 Supplemental Information 1 Information about the KEGG pathway analysis and DMR genes.

10.7717/peerj.13455/supp-2 Supplemental Information 2 COG, GO and KEGG pathway analysis in CG-type DMGs. (A) COG analysis. (B) GO analysis. (C) top KEGG analysis.

10.7717/peerj.13455/supp-3 Supplemental Information 3 KEGG pathway enrichment analysis based on DEGs in twin lamb muscles under feeding conditions.

10.7717/peerj.13455/supp-4 Supplemental Information 4 Author Checklist.

## Additional Information and Declarations

### Data Availability

The following information was supplied regarding data availability: The whole genome methylation sequencing data is available at NCBI: PRJNA814523. The transcriptome sequencing data is available at NCBI SRA: PRJNA659895.
