# Supplementary figures and images for: The impact of different feeds on DNA methylation, glycolysis/gluconeogenesis signaling pathway, and gene expression of sheep muscle
Source: PeerJ. 2022 May 26;10:e13455. doi: 10.7717/peerj.13455 (PMC9148555; doi:10.7717/peerj.13455)

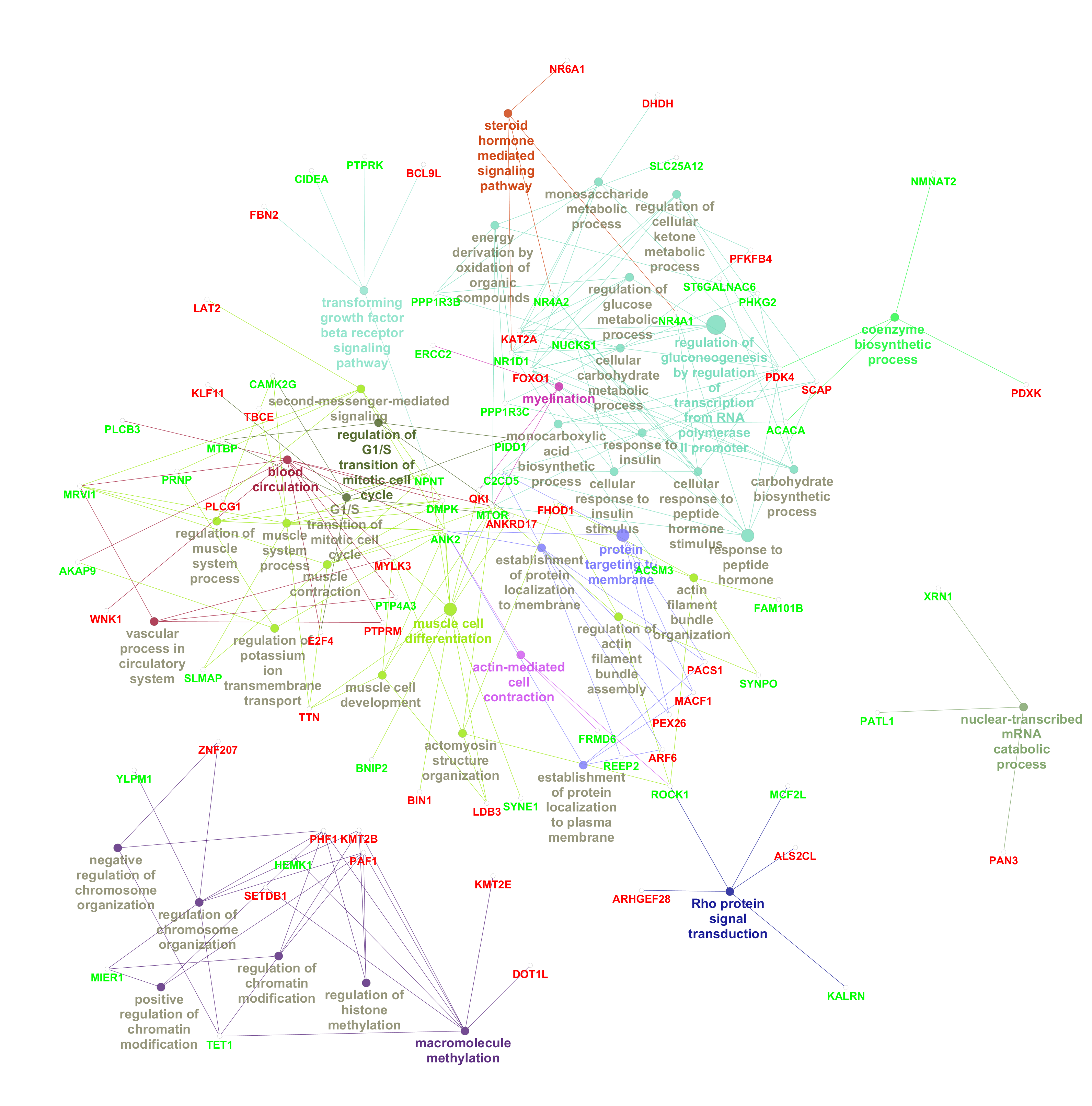

Supplement: Supplemental Information 3 [file peerj-10-13455-s003.png]
